# Supplementary material for: Controlling synthetic membraneless organelles by a red-light-dependent singlet oxygen-generating protein
Source: Nat Commun. 2022 Jun 9;13:3197. doi: 10.1038/s41467-022-30933-0 (PMC9184582; doi:10.1038/s41467-022-30933-0)
Supplement: Supplementary file 1 — Supplementary Information [file 41467_2022_30933_MOESM1_ESM.pdf]

Supplementary Information

Controlling synthetic membraneless organelles by a red-light-dependent singlet oxygen-generating protein

Manjia Li[1], Byung Min Park[1], Xin Dai[2, 3], Yingjie Xu[1, 4], Jinqing Huang[2] and Fei Sun[1, 4, 5, 6*]

[1]Department of Chemical and Biological Engineering, The Hong Kong University of Science and Technology, Clear Water Bay, Kowloon, Hong Kong SAR, China.
[2]Department of Chemistry, The Hong Kong University of Science and Technology, Clear Water Bay, Hong Kong, China.
[3]Laboratory for Synthetic Chemistry and Chemical Biology, Health@InnoHK, Hong Kong Science Park, Hong Kong, China.
[4]Greater Bay Biomedical InnoCenter, Shenzhen Bay Laboratory, Shenzhen 518036, China.
[5]Biomedical Research Institute, Shenzhen Peking University–The Hong Kong University of Science and Technology Medical Center, Shenzhen 518036, China.
[6]HKUST Shenzhen Research Institute, Shenzhen 518057, China.
*Corresponding author. Email: kefsun@ust.hk

This file includes:
Supplementary Figs. 1 to 19
Supplementary Table 1

His6-tag-RGG-Mfp-3-RGG

MKGSSHHHHHHVEASESNQSNNGGSGNAALNRGGRYVPPHLRGGDGGAAAAASAGG
DDRRGGAGGGGYRRGGGNSGGGGGGGYDRGYNDNRDDRDNRGGSGGYGRDRNYED
RGYNGGGGGGGNRGYNNNRGGGGGGYNRQDRGDGGSSNFSRGGYNNRDEGSDNRG
SGRSYNNDRRDNGGDGVDGGGGSELADYYGPKYGPPRRYGGGNYNRYGRRYGGYKG
WNNGWKRGRWGRKYYTSGGGGSLEESNQSNNGGSGNAALNRGGRYVPPHLRGGDG
GAAAAASAGGDDRRGGAGGGGYRRGGGNSGGGGGGGYDRGYNDNRDDRDNRGGSG
GYGRDRNYEDRGYNGGGGGGGNRGYNNNRGGGGGGYNRQDRGDGGSSNFSRGGYN
NRDEGSDNRGSGRSYNNDRRDNGGDG

LvWSCP

INDEEPVKDTNGNPLKIETRYFIQPASDNNGGGLVPANVDLSHLCPLGIVRTSLPYQPGLPV
TISTPSSSEGNDVLTNTNIAITFDAPIWPCPSSKTWTVDSSSEEKYIITGGDPKSGESFFRIE
KYGNGKNTYKLVRYDNGEGKSVGSTKSLWGPALVLNDDDDSDENAFPIKFREVDT

Supplementary Figure 1. Amino acid sequences of RGG-Mfp-3-RGG and LvWSCP.

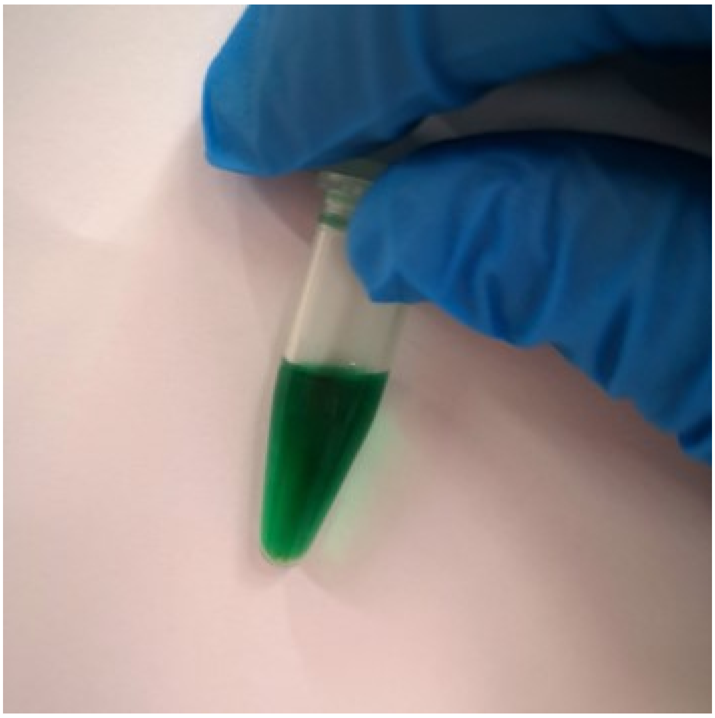

Supplementary Figure 2. Reconstituted chlorophyll-binding WSCP.

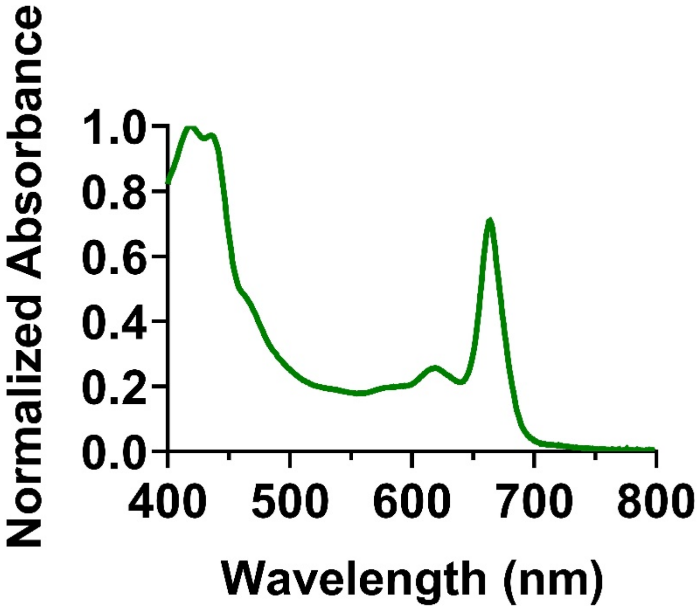

Supplementary Figure 3. Absorbance spectrum of the reconstituted chlorophyll-binding WSCP.

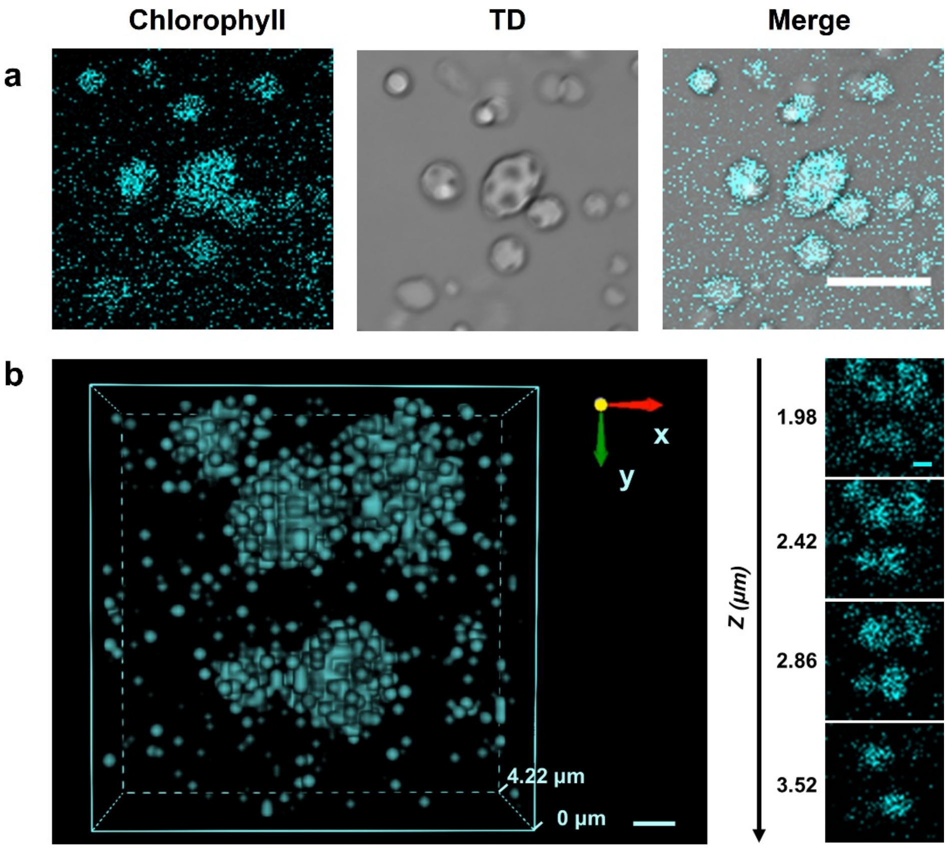

Supplementary Figure 4. Confocal images of RMR/WSCP condensates. a Enrichment of WSCP by RMR condensates. Ex.: 405 nm; Em.: 640 nm. TD, transmitted detector image. Images representative of n = 7. Scale bar: 10 µm. **b** 3D rendering and representative z-slice images of RMR/WSCP condensates. Scale bar: 1 µm. Images representative of n = 4.

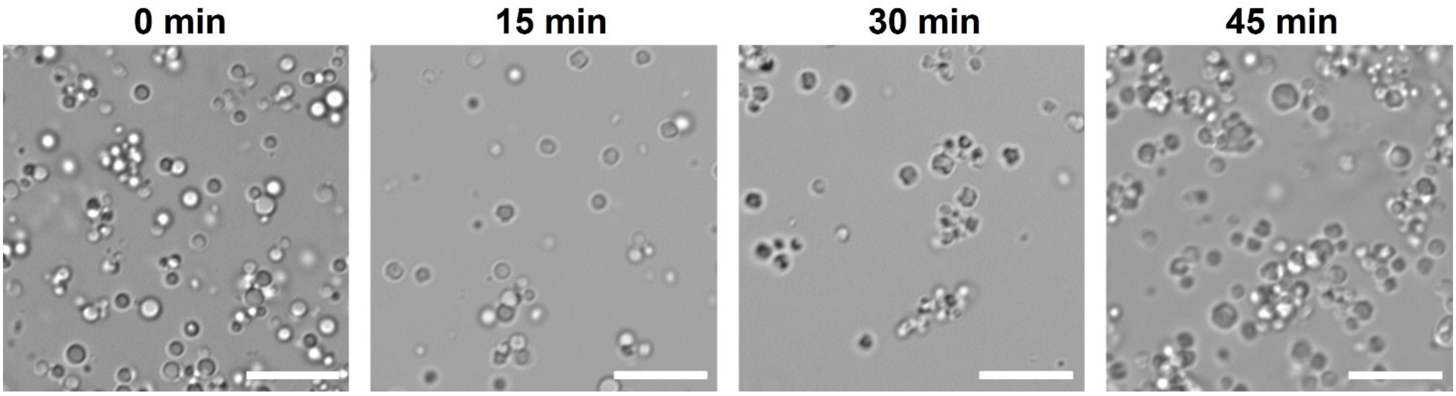

Supplementary Figure 5. Representative images of RMR condensates under red-light illumination with the passage of time. Light intensity: 5 mW/cm^2. Scale bars: 10 μm. Images representative of n = 3.

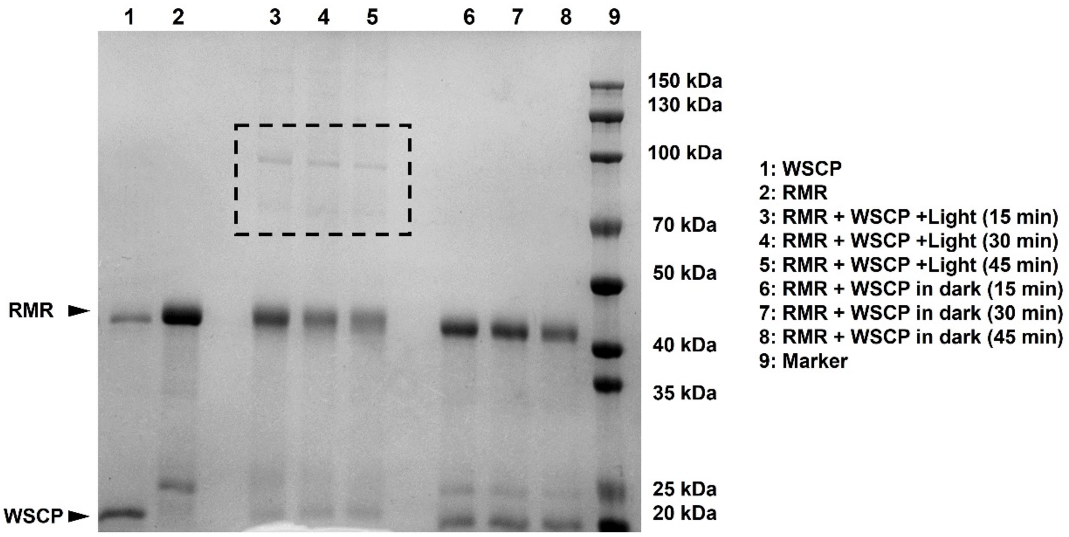

Supplementary Figure 6. SDS-PAGE analysis of RMR/WSCP condensates under light and dark conditions. Some crosslinked products are highlighted in a dash-line box. Images representative of n = 2.

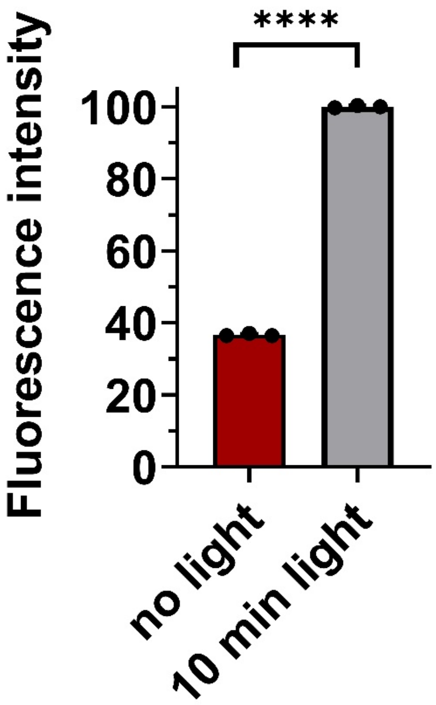

Supplementary Figure 7. Normalized fluorescence intensity of RMR/WSCP before and after red-light illumination ($\lambda_{ex} = \mathbf{280\ nm}$; $\lambda_{em} = \mathbf{400\ nm}$). Data are presented as mean ± SD, (n = 3); two-side t-test, p-value: ****<0.0001(p = 1.74 × 10^{-5}).

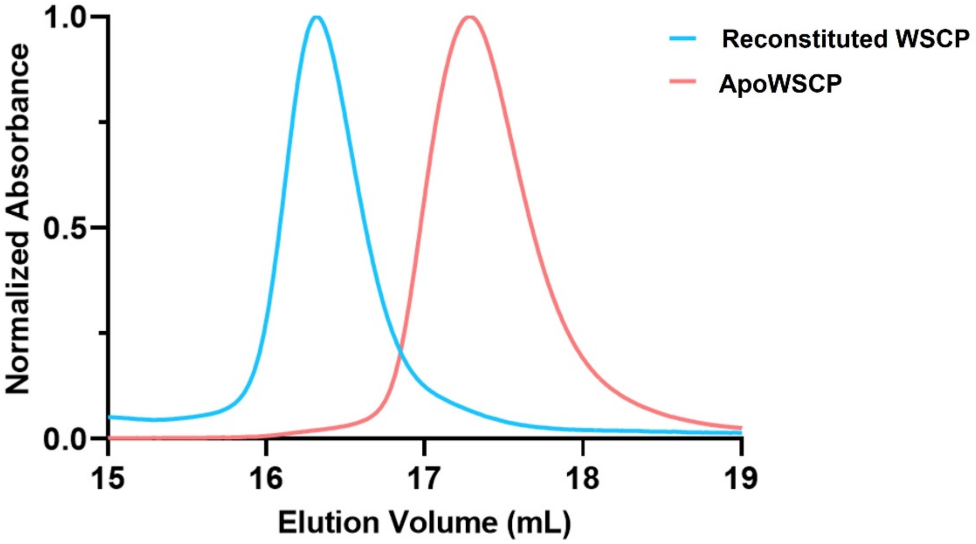

Supplementary Figure 8. SEC analyses of the reconstituted chlorophyll-binding WSCP (tetrameric) and apoWSCP (monomeric).

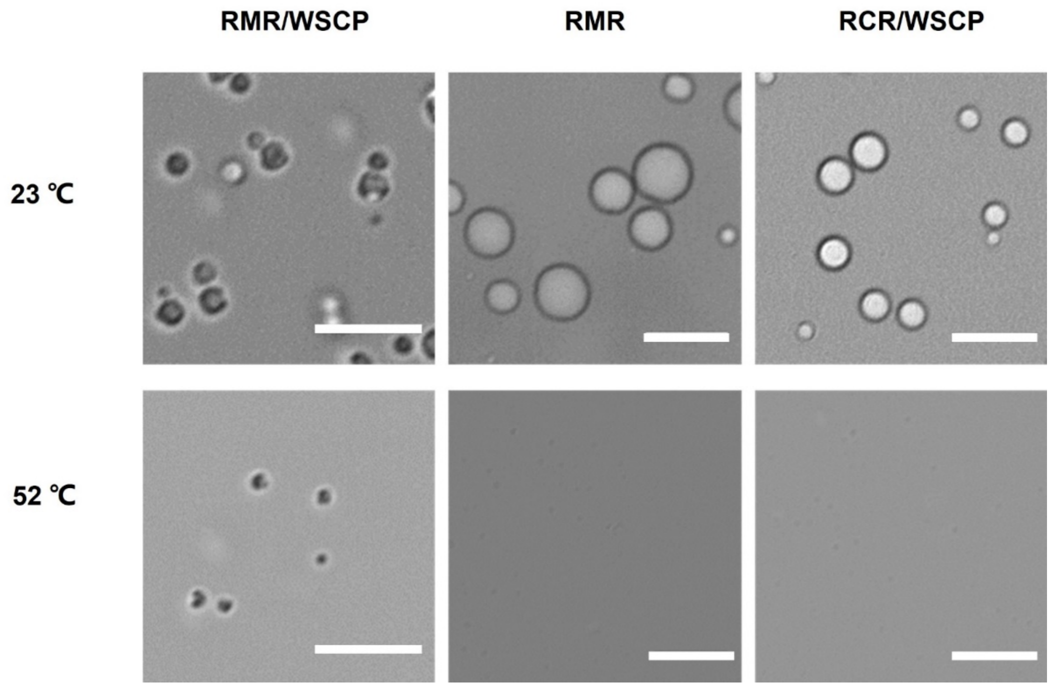

Supplementary Figure 9. Images of RMR/WSCP, RMR, and RCR/WSCP condensates throughout one light-heat cycle. Samples were exposed to red-light (10 mW/cm^2) for 15 min at 23°C, followed by 5-min incubation at 52°C in the dark. RGG-CarH$_C$-RGG (RCR), with the Mfp-3 domain replaced with a globular protein, CarH$_C$, served as a control. Images were taken before and after the 52°C incubation. Scale bars: 10 μm. Images representative of n = 3.

Light intensity: 1 mW/cm^2

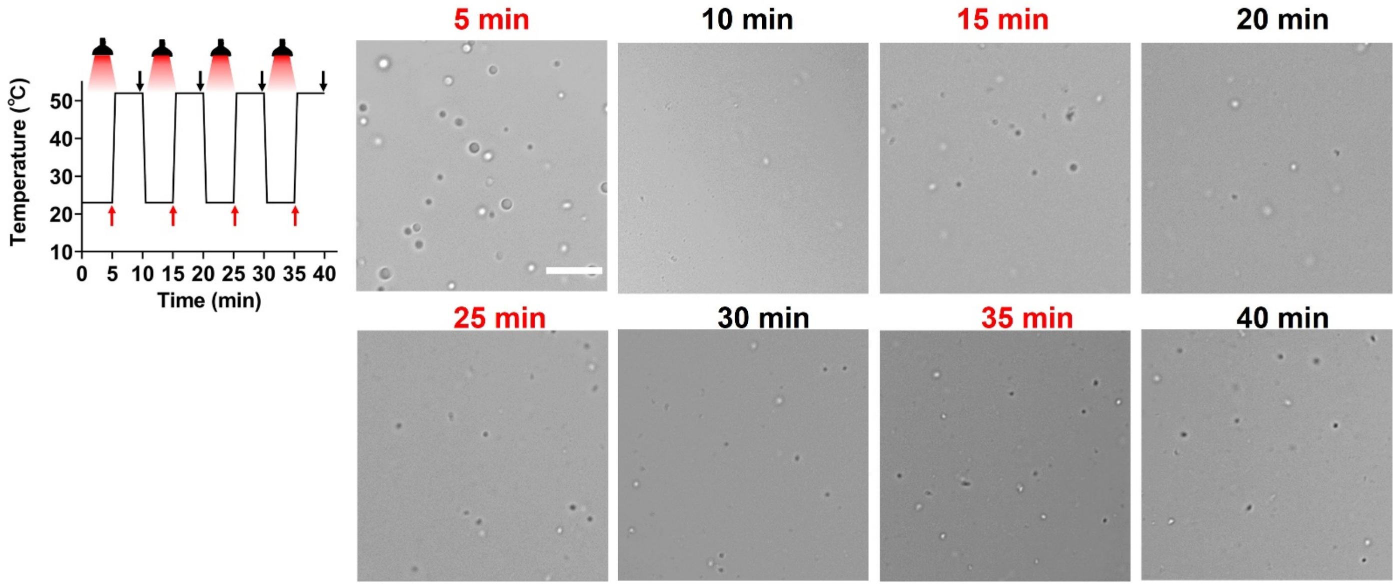

Supplementary Figure 10. Representative images of RMR/WSCP condensates during light-heat cycles at light intensity of 1 mW/cm^2. Samples were collected every 5 min, as indicated by the red and black arrows. Scale bar: 10 μm.

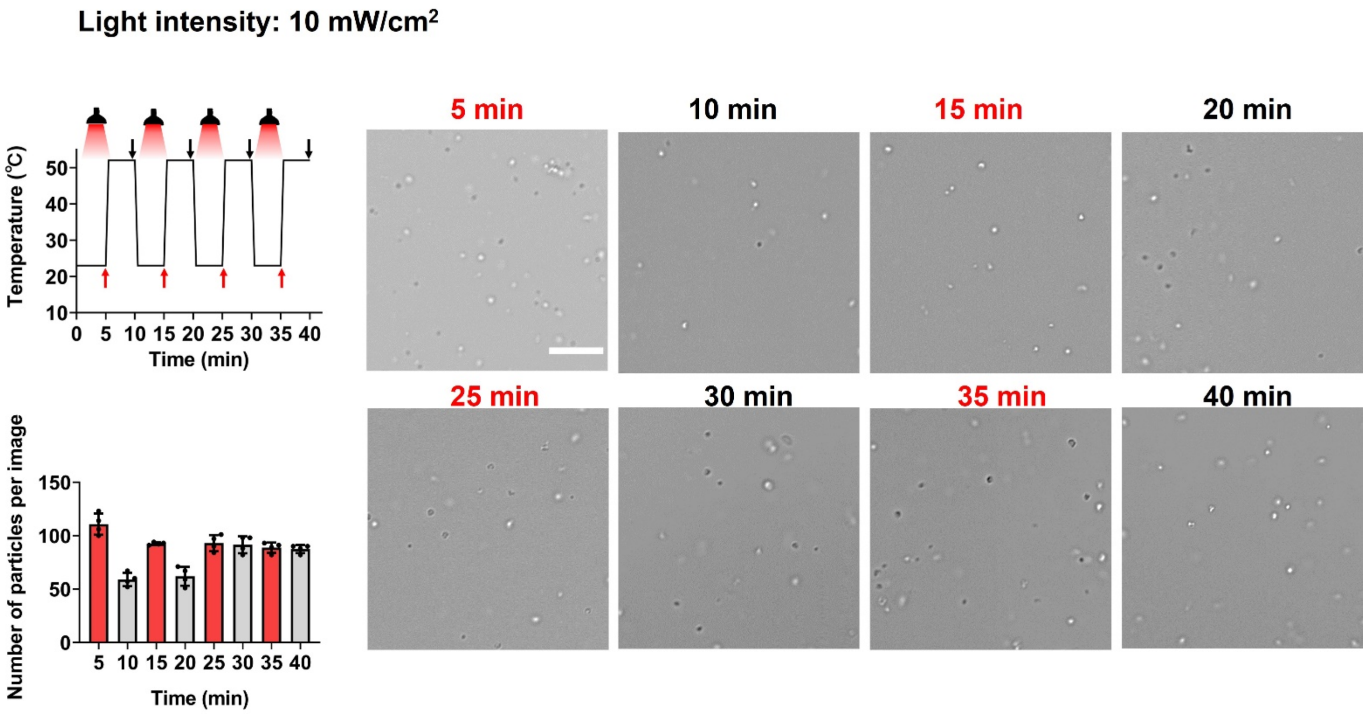

Supplementary Figure 11. Representative images of RMR/WSCP condensates during light-heat cycles at light intensity of 10 mW/cm^2. Samples were collected every 5 min, as indicated by the red and black arrows. The histogram shows the number of condensates per image with the passage of time. Data are presented as mean ± SD. Scale bar: 10 μm.

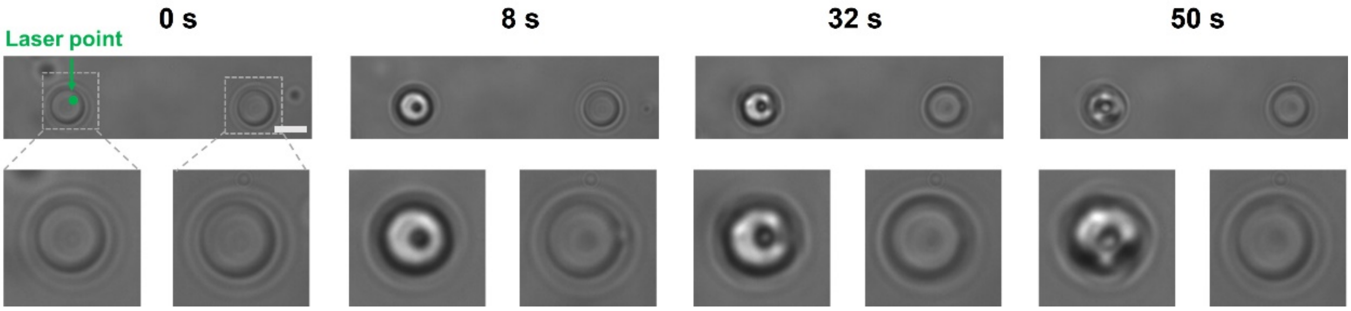

Supplementary Figure 12. Liquid-to-solid phase transition of a trapped RMR/WSCP condensate (*left*) under the irradiation of a triggering laser beam. The concentration of WSCP is 1 μM. Scale bar: 5 μm. Images representative of n = 3.

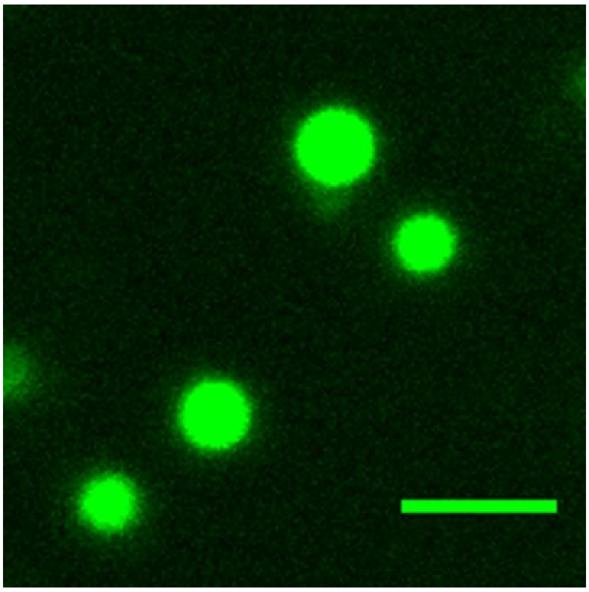

Supplementary Figure 13. Fluorescence micrographs showing GFP-laden RMR condensates in the presence of Zn^{2+} (50 μM). Scale bars: 10 μm. Images representative of n = 3.

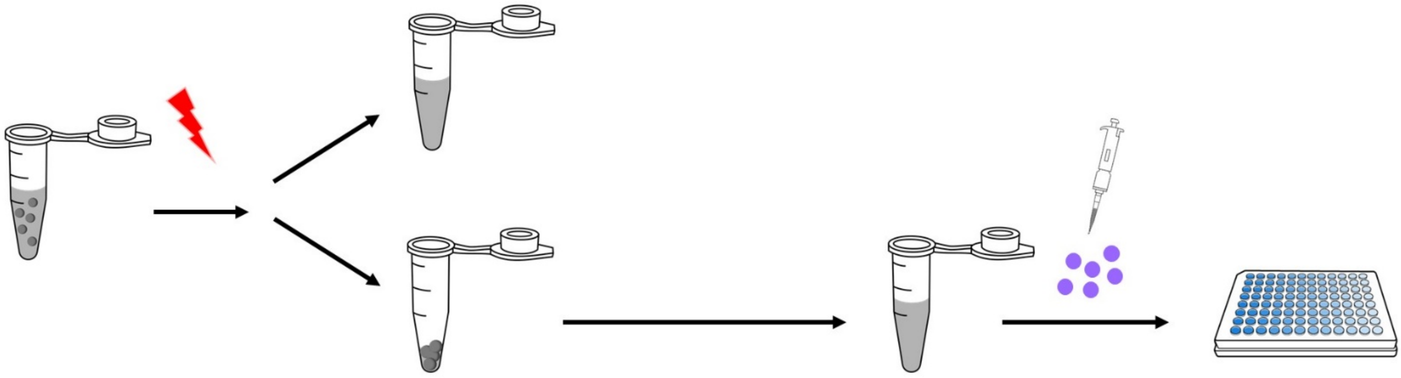

Supplementary Figure 14. Schematic diagram illustrating the workflow of analyzing the red-light controlled catalysis within RMR/WSCP condensates. The RMR/WSCP condensates laden with His6-caspase-3 were exposed to red light, isolated via centrifugation, and then re-suspended in the buffer for the caspase-3 activity assay. The caspase-3 substrate, Z-DEVD-AFC, was subsequently added to initiate the reaction. After 1.5 h, the fluorescence intensity of the reaction mixture was measured.

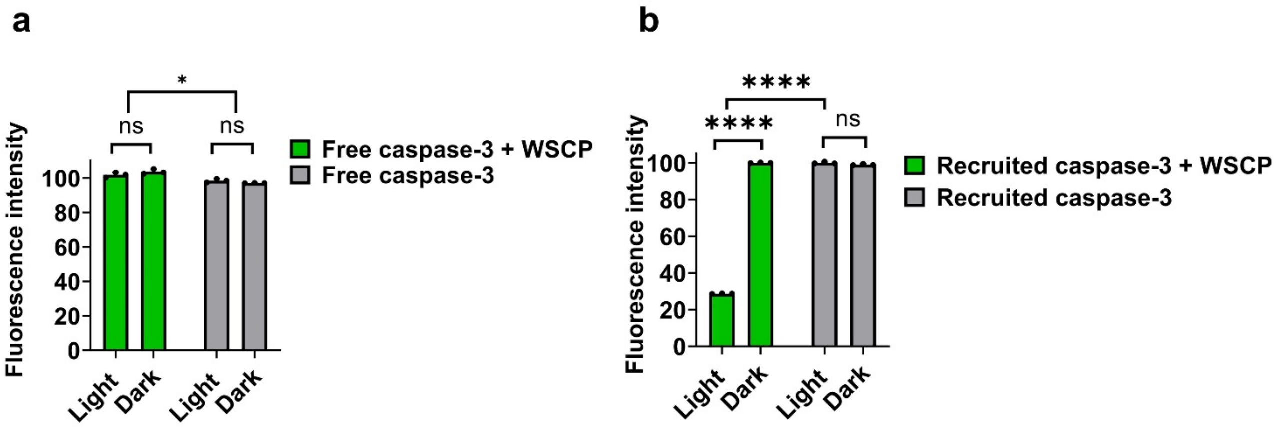

Supplementary Figure 15. Negligible influence of singlet oxygen generated by WSCP on free caspase-3 after 30-min red-light irradiation. Hydrolysis of the fluorogenic substrate for caspase-3, Z-DEVD-AFC, which was accompanied with an increased emission at 500 nm (ex. 405 nm), served as an indicator of the caspase-3 activity. **a** Normalized fluorescence intensities showing the influence of red-light irradiation on free caspase-3 in the solutions in the presence and absence of WSCP. **b** Normalized fluorescence intensities showing the influence of red-light irradiation on bound caspase-3 within RMR condensates in the presence and absence of WSCP. Light intensity: 5 mW/cm^2. Data are presented as mean ± SD (n=3); two-side t-test, p-values: *<0.05 ($p = 0.0341$); ****<0.0001 [$p = 5.61 \times 10^{-7}$ (Light + WSCP *vs* Dark + WSCP); $p = 2.19 \times 10^{-5}$ (Light + WSCP *vs* Light - WSCP)].

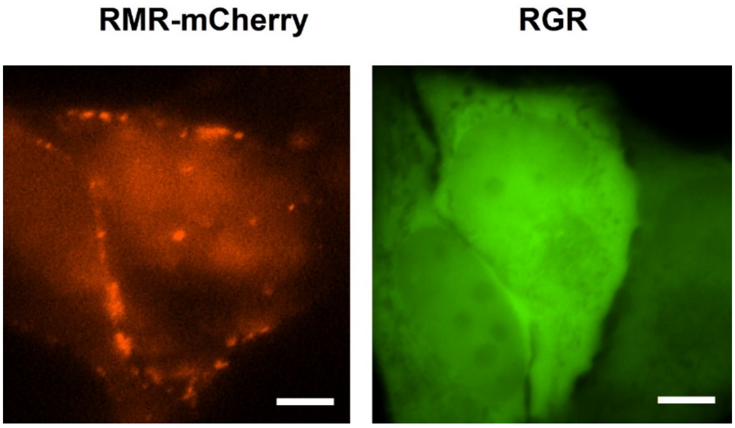

Supplementary Figure 16. RMR-based synthetic membrane-less organelles in Hela cells. Hela cells were transfected with the plasmid harboring the gene encoding RMR-mCherry or RGG-GFP-RGG (RGR). Scale bars: 10 μm. Images representative of n = 3.

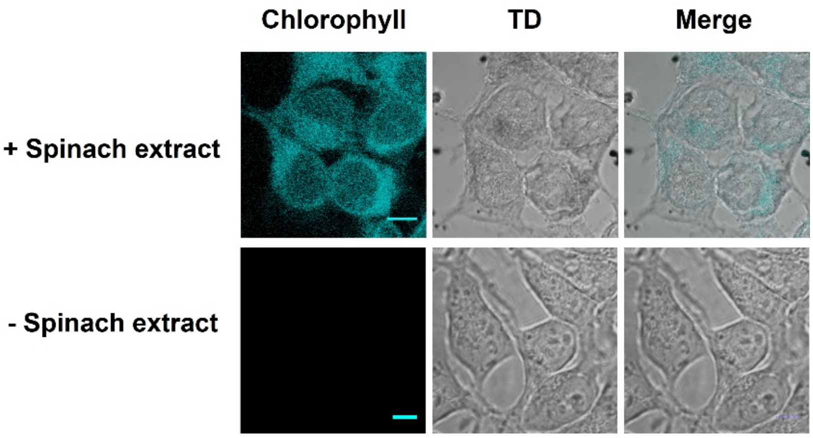

Supplementary Figure 17. Fluorescence images showing the distribution of chlorophyll in HEK293 cells. Cells were cultured in DMEM supplemented with/without spinach extracts for 6 h. TD, transmitted detector image. Scale bars: 10 μm. Images representative of n = 3.

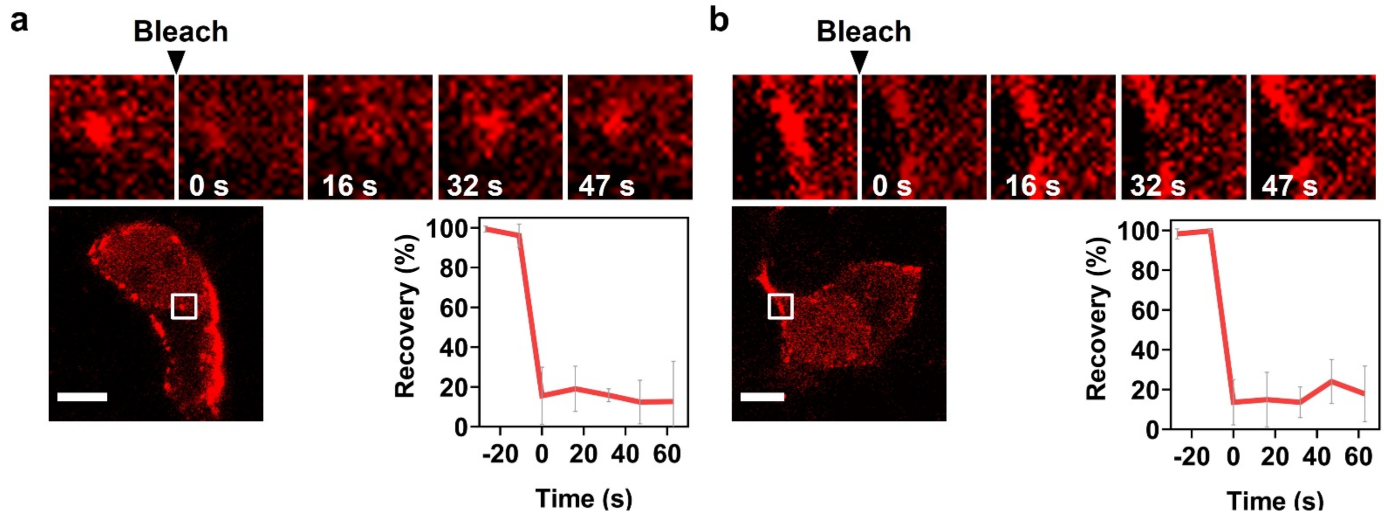

Supplementary Figure 18. FRAP assays of RMR condensates in the cytoplasm (a) and in the outer membrane (b) of HEK293 cells that were harbouring chlorophyll and expressing RMR-mCherry. Scale bars: 10 µm. Data are presented as mean ± SD (n = 5 in **a** and **b**).

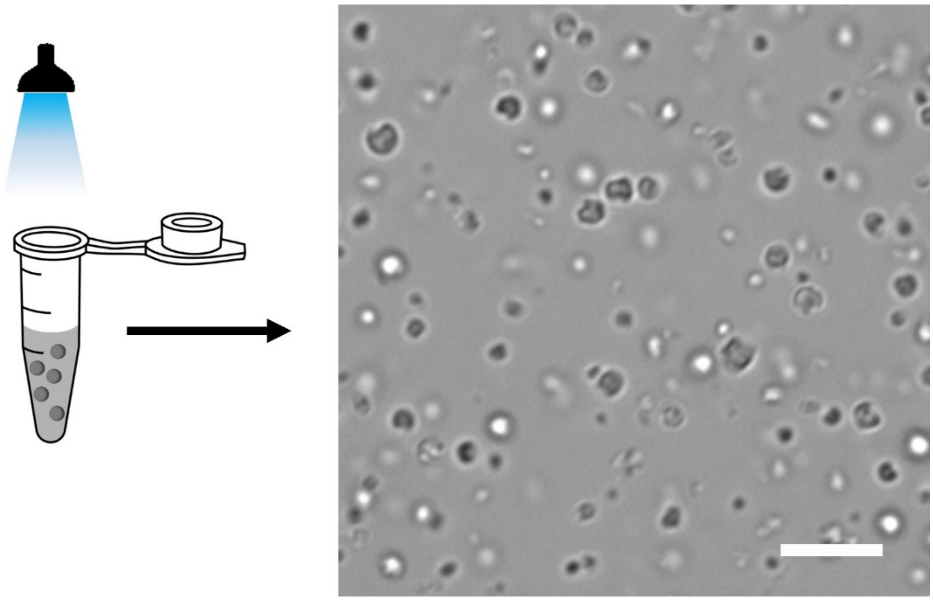

Supplementary Figure 19. Representative image of RMR/WSCP condensates after 10-min blue-light (460 nm) illumination. Light intensity: 10 mW/cm^2. Scale bar: 10 μm. Images representative of n = 3.

Strain	Relevant Characteristics	Source
E. coli DH5α		Stratagene
E. coli BL21 star(DE3)		Invitrogen
**Mammalian cell**	**Relevant Characteristics**	**Source**
HEK293T		ATCC
Hela		ATCC
**Plasmid**	**Relevant Characteristics**	**Source**
pOE80l::*His6-RGG-mfp-3-RGG*	Plasmid for expression of RGG-Mfp-3-RGG	This study
pET22b::*LvWSCP*	Plasmid for expression of LvWSCP	This study
pET21b::*procaspase3*	Plasmid for expression of caspase-3	Prof. A. Clay Clark at North Carolina State University[1]
,	Plasmid for expression of RGG-Mfp-3-RGG-mCherry in mammalian cells	This study
pcDNA::*RGG-gfp-RGG*	Plasmid for expression of RGG-GFP-RGG in mammalian cells	Addgene (Plasmid #124930) [2]
pEGFP::*LvWSCP-egfp*	Plasmid for expression of WSCP-EGFP in mammalian cells	This study

Supplementary Table 1. Bacterial strains, mammalian cells, and plasmids used in this study.

Reference

1. Pop, C. *et al.* Removal of the pro-domain does not affect the conformation of the procaspase-3 dimer. *Biochemistry* **40**, 14224-14235 (2001).
2. Schuster, B.S. *et al.* Controllable protein phase separation and modular recruitment to form responsive membraneless organelles. *Nat Commun* **9**, 2985 (2018).
