# Supplementary material for: Controlling synthetic membraneless organelles by a red-light-dependent singlet oxygen-generating protein
Source: Nat Commun. 2022 Jun 9;13:3197. doi: 10.1038/s41467-022-30933-0 (PMC9184582; doi:10.1038/s41467-022-30933-0)
Supplement: Supplementary file 3 — Description of additional Supplementary File [file 41467_2022_30933_MOESM3_ESM.docx]

**Descriptions of additional supplementary data files**

Supplementary video 1. Representative video showing RMR condensates at 23°C. Scale bar: 1 μm.

Supplementary video 2. Representative video showing RMR condensates in the presence of WSCP at 23°C. Scale bar: 1 μm.

Supplementary video 3. Appearance of an RMR/WSCP condensate at 23 °C under illumination (5 mW/cm2 ) (plays at 7 times speed). Scale bar: 1 μm.

Supplementary video 4. Liquid-to-solid phase transition of the chosen RMR/WSCP condensate (left) under the irradiation of a triggering laser beam. The concentration of WSCP is 5 μM. Scale bar: 5 μm.

Supplementary video 5. Liquid-to-solid phase transition of the chosen RMR/WSCP condensate (left) under the irradiation of a triggering laser beam. The concentration of WSCP is 1 μM. Scale bar: 5 μm.

Supplementary video 6. 3D rendering of HEK293 cells producing RMR-mCherry.
